# Patient Query in Adolescent Testicular Torsion Cases: "Was it Necessary to Mention My Scrotal Pain?"

**DOI:** 10.7759/cureus.47386

**Published:** 2023-10-20

**Authors:** Feride Mehmetoğlu

**Affiliations:** 1 Pediatric Surgery, Dortcelik Children's Hospital, Bursa, TUR

**Keywords:** testicular torsion, adolescent, delayed diagnosis, pain, genital examination, anamnesis, same gender physician

## Abstract

Objective

This study investigates the reasons for the failure to diagnose testicular torsion (TT) in different healthcare facilities (HCFs) and by various physicians.

Method

We retrospectively analyzed all male patients who underwent TT surgery within the adolescent age group between 2015 and 2023. Healthy adolescent patients who initially presented or were referred to an HCF and were subsequently diagnosed at our hospital for TT surgery were analyzed, focusing on why they were not diagnosed earlier.

Results

A total of 11 patients aged 10 to 17 who were surgically confirmed to have TT at our hospital between 2015 and 2023 were analyzed retrospectively. These patients had been admitted to various public and private HCFs due to the sudden onset of symptoms such as abdominal pain, vomiting, fainting, sweating, and walking difficulties during and after working hours. Four patients had previously been admitted to one HCF, while seven had been admitted two to six times to an HCF. All patients were healthy, and all but one had received age-appropriate education. However, only two of them had reported experiencing scrotal pain. Laboratory tests and/or radiological examinations were conducted on eight patients, and nine patients received medical treatment and/or prescriptions. None of the patients were initially diagnosed with acute scrotum.

Conclusion

Despite having unrestricted and free access to well-equipped HCFs, the early diagnosis and treatment of TT are not ensured. Factors contributing to this delay include patients' concealment of, or failure to fully disclose, scrotal complaints, as well as physicians' incomplete history taking and genital examinations. The physician's male gender was not found to contribute to an earlier diagnosis of TT. A mandatory genital examination should be included in the emergency assessment protocol for male patients.

## Introduction

Testicular torsion (TT) is the leading cause of testicular loss. The failure to diagnose TT promptly and accurately can lead to testicular ischemia and subsequent loss. The most common reason for delayed TT diagnosis is the lack of a thorough genitourinary examination. Given its acute nature, clinical examination is crucial for diagnosis [1-3]. Unfortunately, not all patients with TT who are admitted to the emergency department (ED) report testicular pain or noticeable scrotal changes. TT may manifest with abdominal or urinary complaints or walking difficulties [4-7]. However, scrotal findings, particularly varying severity of testicular pain, are consistently present [4]. Therefore, healthcare providers often overlook this differential diagnosis in male patients presenting with non-genital complaints, resulting in a failure to recognize TT [5,6]. In fact, some adolescents experience sexual shyness, leading them to conceal their scrotal complaints and instead report abdominal or inguinal pain, along with other associated symptoms. These patients later admitted that they did not understand the importance of discussing scrotal pain and changes with their families and physicians, often due to feelings of embarrassment [4,6,8,9]. In this study, we aimed to emphasize that incomplete anamnesis and physical examination are the main reasons for late diagnosis of TT, coupled with the reality that atypical presentations of TT are not uncommon.

## Materials and methods

We conducted a retrospective review of all TT cases who presented at the Department of Pediatric Surgery Clinic in a children's hospital from January 2015 to May 2023. Potentially eligible patients were screened for inclusion and exclusion criteria. We excluded patients who were outside the appropriate age range, those unable to adequately verbalize testicular or scrotal changes due to developmental, cognitive, or social challenges, and those who had been primarily diagnosed at our hospital and previous history of scrotal pathologies, including trauma. Only patients within the specified age group, diagnosed, treated, and followed up at our hospital and for whom comprehensive data was accessible were included in the study. We focused on previously healthy adolescent male patients, aged 10 to 17 years, without any intellectual, developmental, or physical disabilities, who were initially admitted or referred to a healthcare facility (HCF) and subsequently underwent surgery where TT was confirmed. The patients underwent TT surgery by a single pediatric surgeon. The patients were identified through a comprehensive review of consecutive cases using digital medical records and paper charts. We analyzed these patients to understand why they were not diagnosed and treated earlier. Data collected included age, medical history, family background, education level, the type and number of HCFs visited, the gender and specialties of the attending physicians, the onset of complaints, and details of the surgical management. As TT cases often have legal implications and are frequently litigated, we meticulously recorded detailed histories, medical files, and operating theater records, including photos and videos, in a timely manner. Additionally, to complete any missing historical data for this study, we contacted patients or their first-degree relatives by phone or in person. The hospital's ethics committee approved this study, and the parents/guardians of the patients provided written and verbal informed consent.

Statistical analysis

Analyses were performed using SPSS version 29.0 (IBM Corp., Armonk, NY, USA). Patient characteristics were reported as n (percent) for categorical variables and as mean ± SD or median (minimum-maximum) for continuous variables, respectively.

## Results

**Figure 1:**
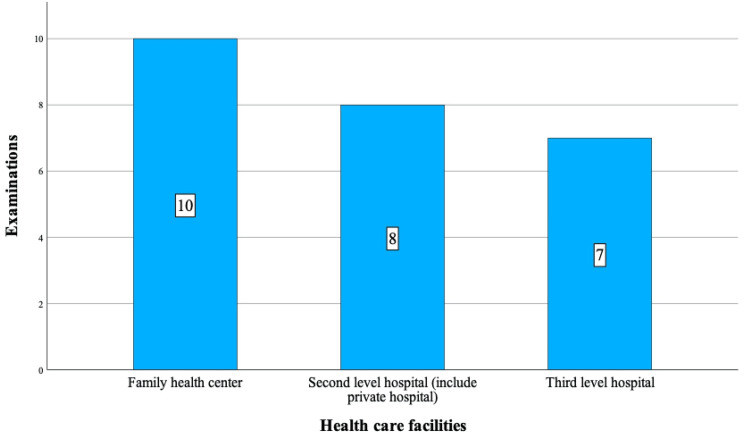
Distribution of patient examinations across different health care facilities.

**Figure 2:**
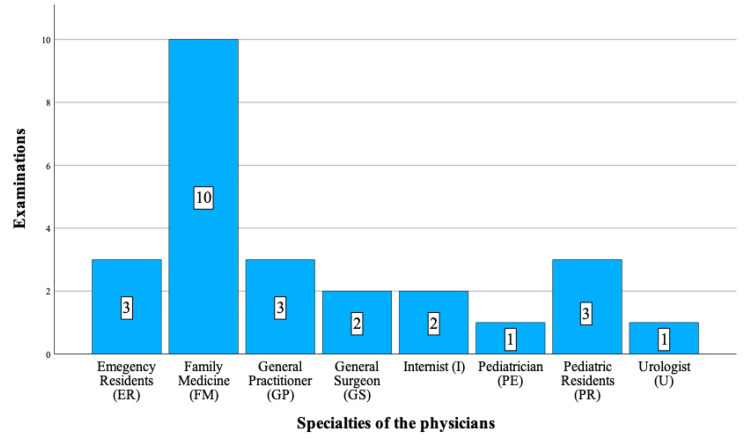
Distribution of examinations according to physicians' specialties.

This eight-year retrospective study involved 11 patients, aged 10 to 17, who underwent surgery for TT. Of these, seven had left-sided TT, and four had right-sided TT. Four of these patients were previously admitted to one HCF, while the remaining seven had visited between two and six different health centers. Notably, three patients had been admitted to the same center twice, and one patient had visited two different centers on two occasions. Patients with complaints of sudden-onset pain (abdominal, back, groin), vomiting, fainting, sweating, mild diarrhea, constipation, urinary complaints, and difficulty walking were examined by various healthcare professionals, including family medicine practitioners, GPs, residents (emergency service, pediatric), and consultant physicians (pediatricians, general surgeons, internists, urologists) at different levels of public and private HCFs, both during and after working hours (Figures 1-2 and Table 1).

**Table 1:** Distribution of HCF groups and physician specialties. HCF: Health care facilities.

Characteristics (N=25)	n (%)
Number of HCF level	
Family health center	10 (40)
Second-level hospital (include private hospital)	8 (32)
Third-level hospital	7 (28)
Specialties of the physicians	
Emergency residents (ER)	3 (12)
Family medicine (FM)	10 (40)
General practitioner (GP)	3 (12)
General surgeon (GS)	2 (8)
Internist (I)	2 (8)
Pediatrician (PE)	1 (4)
Pediatric residents (PR)	3 (12)
Urologist (U)	1 (4)
Gender of the physicians	
Male	18 (72)
Female	7 (28)

**Figure 3:**
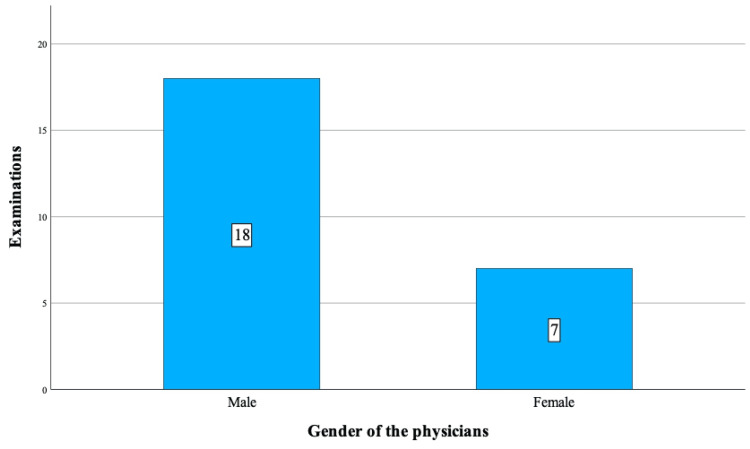
Distribution of examinations by gender of physicians.

All patients were otherwise healthy, with none having a history of undescended testis, acute scrotum, or scrotal pathologies, including trauma. Except for one patient, all attended age-appropriate schooling, with two being bright students, one a national athlete, and one a part-time apprentice. Patients came from diverse socio-economic backgrounds and were either only children or had siblings. However, scrotal changes such as pain, discoloration, or swelling were reported by only two patients. Laboratory tests (blood, urine) and/or radiographic examinations (abdominal X-ray, ultrasound, and CT scan) were performed in eight patients, leading to diagnoses such as vomiting, gastroenteritis, appendicitis, constipation, food poisoning, myalgia, urinary infection, and urolithiasis. Six patients received IV fluids/analgesics/antiemetics/enemas at healthcare facilities, while prescriptions for analgesics/antibiotics were given to six patients. Three patients received both ED treatment and prescriptions. Although there was occasional relief during the time from the first presentation to the surgery of TT, abdominal pain, vegetative symptoms, and other complaints' intensity increased despite various medical treatments (Table 2). In total, 11 patients underwent examinations 25 times (four patients were examined once, four patients twice, one patient three times, one patient four times, and one patient six times), with seven of them examined by female physicians (Figure 3). During our examination, two patients reported complete relief from scrotal pain, while the other three mentioned intermittent pain. However, upon examination, they all exhibited mild to moderate pain upon scrotal palpation. Furthermore, two patients had complained of scrotal changes, but no differential diagnosis of acute scrotum was made during their initial admission to HCFs.

**Table 2:** Distribution of patients’ demographics and clinical findings.

Characteristics (N=11)	n (%)	Mean±SD	Median (Min-Max)
Age (year)		14±2	14 (10-17)
Reporting scrotal findings			
No	9 (81.8)		
Yes	2 (18.2)		
Lab/Rad			
No	3 (27.3)		
Yes	8 (72.7)		
ED medical treatment			
No	5 (45.5)		
Yes	6 (54.5)		
ED medical prescription			
No	5 (45.5)		
Yes	6 (54.5)		
The hour between the onset of the complaints and the admission to our hospital		31±30	17 (4-96)
R/L			
Right	4 (36.4)		
Left	7 (63.6)		
Surgery			
Detorsion (DET)	4 (36.4)		
Detorsion+Orchidopexy (DET+O)	3 (27.3)		
Removal (REM)	2 (18.2)		
Removal+Orchidopexy (REM+O)	2 (18.2)		

All 11 patients underwent surgical exploration, confirming the diagnosis of TT. The decision to perform an orchiectomy was based on the blood supply to the testicle rather than the time elapsed and the degree of torsion. Four patients underwent orchidectomy due to non-salvageable testes, while seven underwent detorsion with orchidopexy. Four patients in this series applied for medicolegal investigation; two of them were patients with orchiectomy. The decision for contralateral orchiopexy was made on a case-by-case basis with advance preoperative family consent and was applied to five of the eleven patients during the same surgery (Table 3).

**Table 3:** Case-based findings and results. HCF: Health care facilities; A: Family health center; B: Second-level hospital (include private hospital); C: Third-level hospital; FM: Family medicine; GP: General Practitioner; PR: Pediatric residents; ER: Emergency residents; PE: Pediatrician; GS: General surgeon; I: Internist; U: Urologist.

Patient No.	Age	Reporting Scrotal Findings	Number of HCF	HCF Level	Specialties of the Physicians	Gender of the Physicians	Lab/Rad	ED Medical Treatment	ED Medical Prescription	The Hour Between the Onset of the Complaints and the Admission to our Hospital	Side	Surgery
1	10	No	1	1-B	1-ER	1-Male	No	No	No	15	Left	Detorsion (DET)
2	12	No	2	1-C	1-PR	1-Male	Yes	No	Yes	8	Left	Detorsion+Orchidopexy (DET+O)
				2-A	2-FM	2-Female						
3	12	No	1	1-A	1-FM	1-Female	No	Yes	No	12	Left	Removal (REM)
4	13	Yes	1	1-C	1-ER	1-Male	Yes	No	Yes	17	Right	Detorsion (DET)
5	14	No	3	1-A	1-FM	1-Male	Yes	Yes	Yes	96	Left	Removal+Orchidopexy (REM+O)
				2-C	2-PR	2-Female						
				3-C	3-I	3-Male						
6	14	No	2	1-B	1-PE	1-Male	Yes	Yes	No	54	Right	Removal (REM)
				2-B	2-PR	2-Male						
7	15	No	2	1-C	1-U	1-Female	Yes	No	No	6	Left	Detorsion+Orchidopexy (DET+O)
				2-A	2-FM	2-Male						
8	15	No	4	1-A	1-FM	1-Male	Yes	Yes	Yes	41	Right	Removal+Orchidopexy (REM+O)
				2-B	2-GS	2-Male						
				3-A	3-FM	3-Male						
				4-A	4-FM	4-Male						
9	16	Yes	2	1-B	1-GP	1-Female	Yes	No	Yes	19	Right	Detorsion (DET)
				2-A	2-FM	2-Male						
10	17	No	6	1-A	1-FM	1-Male	Yes	Yes	Yes	70	Left	Detorsion (DET)
				2-B	2-GP	2-Female						
				3-C	3-ER	3-Female						
				4-A	4-FM	4-Male						
				5-B	5-GP	5-Male						
				6-B	6-I	6-Male						
11	17	No	1	1-C	1-GS	1-Male	No	No	No	4	Left	Detorsion+Orchidopexy (DET+O)

## Discussion

TT is an emergency condition requiring early diagnosis and surgery to salvage the involved testis in order to preserve testicular fertility. The diagnosis of TT can be preliminarily established via a complete physical examination. Several factors associated with delayed diagnosis or misdiagnosis of TT increase the possibility of failure to save the affected testicle. Patient-specific factors contributing to delayed presentation may include fear, embarrassment, particularly among adolescent children, reduced pain sensitivity, or deficient communication abilities, particularly among very young or neurologically impaired patients [5,10]. External factors leading to delayed diagnosis or treatment may include inadequate healthcare system infrastructure, lack of clinical expertise among physicians, or misjudgment of the condition by examining healthcare professionals [1,5,11]. The reasons behind late diagnosis in our patient cohort included adolescent sexual embarrassment and a lack of awareness regarding TT. Notably, none of our patients presented with any mental or physical disorders. An external factor contributing to the delay was the omission of a comprehensive examination by physicians, hindering the attainment of a differential diagnosis for TT. Medical history acquisition should also be undertaken directly from the patient, with age-appropriate dialogue, while minimizing the influence of family or caregivers [12]. In some instances, parents do not explain the complete narrative, including the duration of complaints, administered medications, or the use of hot and cold packs at home. Thus, obtaining patient anamnesis without the presence of individuals who brought the patient to the hospital, with a chaperone, or positioning them so that the patient cannot perceive facial expressions or other nonverbal cues can prove effective. Moreover, a distinct medical history can be gleaned without blaming the patient or their family for the delayed admission to the HCF [5]. When queried as to why they failed to report scrotal findings, patients often asked, "Was it necessary to mention my scrotal pain? I noticed scrotal pain, redness, and swelling but deemed it unnecessary to disclose, or I failed to recognize scrotal discomfort and dismissed it, or I hesitated to discuss it." Notably, all patients had internet access, although only one acknowledged conducting online research on scrotal changes, while others may have abstained. Some patients' symptoms had initiated during boarding school or while visiting relatives. Yet, they refrained from reporting their complaints due to uncertainty regarding the appropriate action or out of reticence. Therefore, it is imperative to educate children on expressing changes and complaints related to their genital area at home, in educational institutions, sports clubs, camps, and similar settings [13]. Developmental, cognitive, or social disorders were explored as potential reasons for delayed presentation; however, none of our patients had a history of such conditions [5,10]. All patients were in sound physical and mental health and received age-appropriate education. Additionally, prior scrotal pathologies and trauma were contemplated as etiological factors for TT [7,14]. Nevertheless, none of our patients had a history of scrotal disease, trauma, or complaints that might have prompted mention of genital region issues during history-taking by their families and patients. Since complaints in childhood often include problems such as cough, fever, vomiting, and abdominal pain, the unusualness of genital complaints for the adolescent patient may also be a factor. Families and caregivers typically lack knowledge about TT despite the ready availability of health information from various sources. It is evident that the general awareness of TT is deficient, with most individuals unaware that TT can occur, potentially leading to testicular loss. This deficiency in awareness appears to be true for all levels of society and educational ability, as in our patients' families [5,8,15-18]. In comparison to families whose children underwent surgery for other reasons, our families demonstrated erratic behavior when confronted with the possibility of testicular loss due to delay. Some expressed self-blame or blamed the patient, while most held physicians responsible, threatening legal action [13].

Numerous studies have examined TT pain from various angles. Although testicular pain is a common presentation within the emergency setting, adolescent patients may sometimes struggle to discern or localize their pain and may feel too embarrassed to admit to testicular discomfort [4]. One case report highlighted a delayed TT diagnosis in a patient who was assessed for neurological deficits due to the absence of pain [11]. Another study focused on TT pain, describing the resolution of pain as the "honeymoon period" [9]. Equally, five of our patients reported reduced pain intensity or relief. Children and adolescents are inclined to conceal or downplay scrotal pain [5]. Our observation suggests that these patients may have only experienced decreased pain, as they exhibited pain upon palpation during our examination. The elapsed time and the treatments applied before their admission to our hospital may have also played a role in the honeymoon period. Typically, pain, vegetative symptoms, and scrotal changes prompt timely consultation with a primary care physician or a visit to the ED [3]. Nevertheless, many cases are not diagnosed promptly. Atypical TT presentations can occur in adolescent patients, manifesting with nonspecific symptoms that challenge diagnosis and may lead to delayed treatment, increasing the risk of complications. Acute abdominal pain can present as the initial and sole symptom of TT in young males, and TT can mimic various urologic and intestinal emergencies [4,5,19]. The detailed anamnesis of our patients revealed that, although the order and severity of their complaints varied, each exhibited diverse presentations of TT, including abdominal and/or inguinal pain, nausea and/or vomiting, an urge to urinate, and scrotal pain. Their initial symptoms typically emerged consecutively within 1-3 hours, with some patients unable to discern which symptom appeared first. In our study, four patients were misdiagnosed with urinary infection, and three received enemas due to a suspicion of constipation. Another common factor contributing to delayed TT diagnosis is the multitude of potential causes for acute scrotal issues [2,7]. Since examinations of the scrotal region were either completely overlooked or not documented in our series, none of the patients who presented to us were diagnosed with acute scrotum. This oversight was attributed to the omission of inquiries about scrotal complaints during admission and patients' unwillingness to provide such information. In this series, we analyzed patients who were admitted to an HCF on more than one occasion (with an average of 2.2 times). Similarly, the literature documents multiple admissions to the same or different centers [4,5,9,20]. The litigious nature of TT may lead to frequent patient transfers, primarily due to a lack of pediatric surgeons and color Doppler sonography for precise diagnosis [1,9]. Numerous studies report that children with testicular pain often initially encounter junior physicians, GPs, or ED physicians before being evaluated by a urologist or arriving at a tertiary care center, frequently surpassing the critical early window of 6 hours and resulting in delayed TT diagnosis [6,10]. However, in our series, senior physicians, including urologists, also evaluated some patients who were admitted to a tertiary center during TT's golden hours. The preference for a specific gender of the physician proves highly relevant in genitourinary issues [21,22]. The gender of the examining physician can significantly influence the success of adolescent healthcare interactions [23]. Nevertheless, the male gender of a physician did not appear to impact the early diagnosis of TT in our patients, with 19 out of 25 physicians (76%) being male. Our patients did not specifically disclose their symptoms to physicians of the same gender to discuss their scrotal complaints.

Various scores and treatment algorithms have been proposed in the literature to aid in the early diagnosis of TT [1,19,24]. However, the unique clinical and treatment characteristics of each patient in our study, along with variations in the timing of presentation and treatment, precluded the establishment of a standardized scheme. Furthermore, it should be noted that families and patients may consciously or unconsciously misrepresent the exact onset time of symptoms. Consequently, especially in cases of delayed presentation, individual evaluation should take precedence over scoring systems. Thus, a comprehensive medical history should be elicited, and a meticulous scrotal examination should be performed on all male patients who are admitted for acute issues to the HCF.

The literature suggests a critical 4-8-hour, generally 6-hour window as golden vital hours for testicular salvage [7]. Conversely, cases have been documented where salvage occurred after two days [11]. In our series, one patient underwent detorsion after 70 h and maintained testicular viability, albeit with a reduction in size compared to the contralateral testicle. Moreover, a study reported that patients with early salvage surgery for TT may develop testicular atrophy and not regain normal testicular function [6]. Therefore, we think that the decision for orchiectomy should be more restrained.

TT typically manifests with sudden, intense scrotal pain, often accompanied by vegetative symptoms. This combination should prompt patients to seek medical attention [1]. However, our case series underscores that this is not always the case. TT may not always commence with severe pain, the intensity of pain may vary, and the testicle can maintain its vitality for a longer duration. In contrast to the notion that the absence of scrotal pain in most cases rules out acute TT, our experience suggests that pain intensity may fluctuate, and patients may withhold pain disclosure due to embarrassment. Studies have identified differences in symptoms and medical histories between delayed and acute TT presentation cases. Our study also aligns with these findings, illustrating that patients' symptoms and signs vary widely. The classic presentation of TT does not consistently manifest, and atypical symptoms and signs are not rare.

For decades, TT has been associated with the most common malpractice claims worldwide [20]. Remarkably, despite advancements in healthcare system infrastructure, TT continues to be a predominant cause of malpractice litigation globally [1,25]. Technological progress has not helped missed diagnoses, as the delayed diagnosis of adolescent TT is not solely attributable to inadequately equipped HCFs. Instead, the primary factors contributing to delays are inadequate history taking, insufficient physical examination, and limited physician-patient communication.

Limitations

One limitation of this study pertains to the potential information bias originating from patients and their families. We had limited access to outside HCF records. Our hospital offers round-the-clock emergency pediatric surgery services to a broad geographic area. Furthermore, the reason why patients in this series were admitted to multiple HCFs in a short timeframe is that they have the freedom to seek care at any level of public HCF without incurring charges, and there is a prevalence of private HCFs in the region. Additionally, there is a lack of a specific referral chain.

## Conclusions

Accessibility to a well-equipped health center at vital hours does not always provide early diagnosis and treatment of testicular torsion. The reasons for the delay are the lack of a thorough genital examination by physicians, adolescents’ unawareness about testicular torsion and atypical presentation of the testicular torsion.

Pre-adolescent and adolescent boys should receive information about TT as part of their school health curricula. Families of male patients should also receive information about TT from their family physicians. Genital examination should be conducted on all male patients seeking care at an HCF for acute issues, regardless of whether their complaints involve the genitalia. The importance of genital examination should be emphasized to physicians during the training.
